# Sebetralstat for on-demand treatment of hereditary angioedema: A pooled analysis of placebo-controlled clinical trials^☆^

**DOI:** 10.1016/j.waojou.2026.101401

**Published:** 2026-06-09

**Authors:** Emel Aygören-Pürsün, Danny M. Cohn, Nancy Agmon-Levin, Aleena Banerji, Jonathan A. Bernstein, Paula Busse, Mauro Cancian, Eunice Dias de Castro, Henriette Farkas, Vesna Grivcheva-Panovska, David Hagin, Roman Hakl, Daisuke Honda, Aharon Kessel, Tamar Kinaciyan, IFAAD, Marcin Kurowski, H. Henry Li, Ramon Lleonart, Hilary J. Longhurst, William R. Lumry, Markus Magerl, Inmaculada Martinez-Saguer, Isaac Melamed, Fotis Psarros, Sinisa Savic, Daniel F. Soteres, Maria Staevska, Marcin Stobiecki, H. James Wedner, Andrea Zanichelli, James Hao, Ketan Patil, Matthew Iverson, Samuel Owiredu-Yeboa, Michael D. Smith, Christopher M. Yea, Paul K. Audhya, Marc A. Riedl, Sorena Kiani-Alikhan, Marcus Maurer

**Affiliations:** a University Hospital Frankfurt, Goethe University, Frankfurt, Germany; b Amsterdam UMC Location AMC, Amsterdam, Netherlands; c Clinical Immunology, Angioedema and Allergy Institute, The Center for Autoimmune Diseases, Sheba Medical Center, Tel Aviv, Israel; d Professor of Medicine, Harvard Medical School, Clinical Director, Allergy and Immunology, Massachusetts General Hospital, Boston, Massachusetts, United States; e University of Cincinnati College of Medicine, Division of Rheumatology, Allergy and Immunology, Advanced Allergy Services and Bernstein Clinical Research Center, Cincinnati, Ohio, United States; f Icahn School of Medicine at Mount Sinai, New York, New York, United States; g Departmental Unit of Allergology, University Hospital of Padua, Padua, Italy; h Allergy and Clinical Immunology Department, Centro Hospitalar Universitário de S. João EPE, Porto, Portugal; i Public Health and Forensic Sciences and Medical Education Department, Faculty of Medicine, University of Porto, Porto, Portugal; j Hungarian Angioedema Center of Reference and Excellence, Department of Internal Medicine and Haematology, Semmelweis University, Budapest, Hungary; k ACARE North Macedonia, PHI University Clinic of Dermatology, Ss Cyril and Methodius Skopje University, Skopje, Republic of North Macedonia; l Allergy and Clinical Immunology, Tel Aviv Sourasky Medical Center, Tel Aviv, Israel; m Department of Clinical Immunology and Allergology, St. Annés University Hospital in Brno, and Faculty of Medicine, Masaryk University, Brno, Czech Republic; n Department of Nephrology, Graduate School of Medicine, Chiba University, Chiba, Japan; o Division of Allergy and Clinical Immunology, Bnai Zion Medical Center, Haifa, Israel; p Department of Dermatology, Medical University of Vienna, Vienna, Austria; q Department of Immunology and Allergy, Medical University of Łódź, Łódź, Poland; r Institute for Asthma and Allergy, Chevy Chase, Maryland, United States; s Allergology Department, Bellvitge University Hospital, Barcelona, Spain; t Department of Medicine, University of Auckland and Department of Immunology, Auckland City Hospital, Auckland, New Zealand; u AARA Research Center, Dallas, Texas, United States; v Angioedema Center of Reference and Excellence (ACARE), Institute of Allergology, Charité – Universitätsmedizin Berlin, corporate member of Freie Universität Berlin and Humboldt-Universität zu Berlin, Berlin, Germany; w Fraunhofer Institute for Translational Medicine and Pharmacology ITMP, Immunology and Allergology, Berlin, Germany; x Hemophilia Center Rhine Main, Frankfurt, Germany; y IMMUNOe Research Center, Centennial, Colorado, United States; z Allergy Department, Naval Hospital, Athens, Greece; aa Leeds Institute of Rheumatic and Musculoskeletal Medicine, University of Leeds, Leeds, United Kingdom; bb Asthma & Allergy Associates, Colorado Springs, Colorado, Department of Internal Medicine, Division of Allergy and Immunology, University of Colorado Health Sciences Center, Denver, CO, United States; cc Department of Allergology, Medical University of Sofia, Clinic of Allergology, University Hospital "Alexandrovska” Sofia, Sofia, Bulgaria; dd Department of Clinical and Environmental Allergology, Jagiellonian University Medical College, Krakow, Poland; ee Division of Allergy and Immunology, John T. Milliken Department of Medicine, Washington University School of Medicine, St. Louis, Missouri, United States; ff Operative Unit of Medicine, Angioedema Center, IRCCS Policlinico San Donato, San Donato Milanese, Milan, Italy; gg Department of Biomedical Science for Health, University of Milan, Milan, Italy; hh KalVista Pharmaceuticals, Inc., Framingham, Massachusetts, United States; ii KalVista Pharmaceuticals, Inc., Salisbury, United Kingdom; jj Division of Allergy and Immunology, University of California – San Diego, La Jolla, California, United States; kk Royal Free London NHS Foundation Trust, London, United Kingdom

**Keywords:** Hereditary angioedema, Sebetralstat, Early treatment, Efficacy, On-demand treatment

## Abstract

**Background:**

Clinical trial designs evaluating on-demand therapies for hereditary angioedema attacks have evolved in response to changes in treatment guidelines. Sebetralstat, an oral plasma kallikrein inhibitor, was evaluated in 2 randomized, placebo-controlled clinical trials, which instructed early treatment of attacks with no minimum severity requirement.

**Objective:**

Characterize the efficacy and safety of sebetralstat by pooling data from phase 2 and 3 trials.

**Methods:**

This pooled analysis included participants (phase 2, aged ≥18 years; phase 3, aged ≥12 years) who received ≥1 dose of study drug (phase 2, sebetralstat 600 mg or placebo; phase 3, sebetralstat 300 mg or 600 mg, or placebo). Efficacy outcomes included times to beginning of symptom relief within 12 h, reduction in severity within 12 h, and complete attack resolution within 24 h. *P* values were not adjusted for multiplicity.

**Results:**

377 attacks were treated: 87 with sebetralstat 300 mg; 151 with sebetralstat 600 mg; 139 with placebo. Median (interquartile range) time to treatment was 32.5 min (8.0–94.0). Baseline severity was rated as “Mild” (46.2%), “Moderate” (40.6%), or “Severe”/“Very Severe” (12.7%). Compared with placebo, time to beginning of symptom relief was faster with sebetralstat (300 mg; 600 mg [*P* = 0.0001; *P* < 0.0001]), as was reduction in severity (*P* = 0.0038; *P* < 0.0001) and complete attack resolution (*P* = 0.0021; *P* < 0.0001). Median time to beginning of symptom relief was 1.6 h (0.8–7.0) and 1.8 h (1.0–4.3) with sebetralstat 300 mg and 600 mg, respectively, and 8.3 h (1.5 to >12) with placebo. Sebetralstat had a safety profile comparable to placebo.

**Conclusion:**

Across phase 2 and 3 clinical trials, sebetralstat enabled early treatment, provided effective symptom relief versus placebo, and was well tolerated, regardless of attack location or baseline severity.

**Clinical trial registration:**

ClinicalTrials.gov Identifier NCT04208412, registered on 2019-07-02; ClinicalTrials.gov Identifier NCT05259917 (KONFIDENT), registered on 2022-02-22.

## Introduction

Hereditary angioedema (HAE) is a rare genetic disease characterized by recurrent and unpredictable attacks of mucosal and subcutaneous tissue swelling,^1^^,^^2^ which can be life-threatening if involving the airway.^3^ Most cases of HAE are caused by either C1 inhibitor (C1INH) deficiency (HAE-C1INH-Type1) or dysfunction (HAE-C1INH-Type2).^2^

Historically, HAE treatment guidelines recommended treating attacks based on location and severity; however, these recommendations have evolved over time^4^^,^^5^ based on primary clinical trial results, post hoc analyses, and evidence from real-world studies of on-demand therapies. Current guidelines recommend patients: 1) consider treatment of all attacks, regardless of severity or anatomic location; 2) administer on-demand treatment as early as possible to minimize morbidity and mortality; 3) always carry adequate on-demand medication for at least 2 attacks, because the timing and progression of HAE attacks are unpredictable.^2^ International patient survey data have demonstrated that intravenous and subcutaneous on-demand therapies can result in hesitancy stemming from the risk of injection-site reactions and present logistical challenges (eg, storing, carrying, preparing, finding a discreet and hygienic location); these factors can result in delayed treatment.^6^, ^7^, ^8^, ^9^, ^10^ Late or withheld treatment results in suboptimal clinical outcomes,^2^^,^^11^ therefore it is vital to expedite time to administration to halt the progression of attacks and achieve early symptom relief and attack resolution.

Sebetralstat, a plasma kallikrein inhibitor, was the first orally administered therapy to be evaluated in phase 2 (NCT04208412) and 3 (NCT05259917) clinical trials for on-demand treatment of HAE attacks.^12^^,^^13^ As of November 2025, it has been approved by regulatory bodies in the United States, the European Union, the United Kingdom, Switzerland, and Australia for the treatment of acute attacks of HAE in patients aged 12 years and older.^14^, ^15^, ^16^, ^17^, ^18^ The phase 2 randomized trial enrolled 68 adults with HAE-C1INH-Type1 or 2 who treated 113 attacks with a single dose of sebetralstat 600 mg or placebo, with a median time of 30 min (interquartile range [IQR] 21–50) from recognition of attack to treatment. Sebetralstat was well tolerated and significantly prolonged time to administration of conventional treatment and reduced time to beginning of symptom relief compared with placebo.^12^ In the phase 3 randomized trial, 110 adults and adolescents with HAE-C1INH-Type1 or 2 treated 264 attacks with sebetralstat 300 mg, 600 mg, or placebo, with a median time of 41 min (IQR 6–140) from onset to treatment administration.^13^ Both sebetralstat doses significantly shortened time to beginning of symptom relief, reduction in attack severity, and complete attack resolution versus placebo, with no notable safety signs.

Rare diseases present unique challenges in clinical research, including small patient populations and limited resources. Pooled analyses offer a valuable strategy to overcome these challenges. We present findings of a pooled analysis of the phase 2 and 3 trials of sebetralstat for on-demand treatment of HAE attacks, including novel post hoc analyses made possible with this larger dataset.

## Methods

### Trial designs and participants

**Fig. 1:**
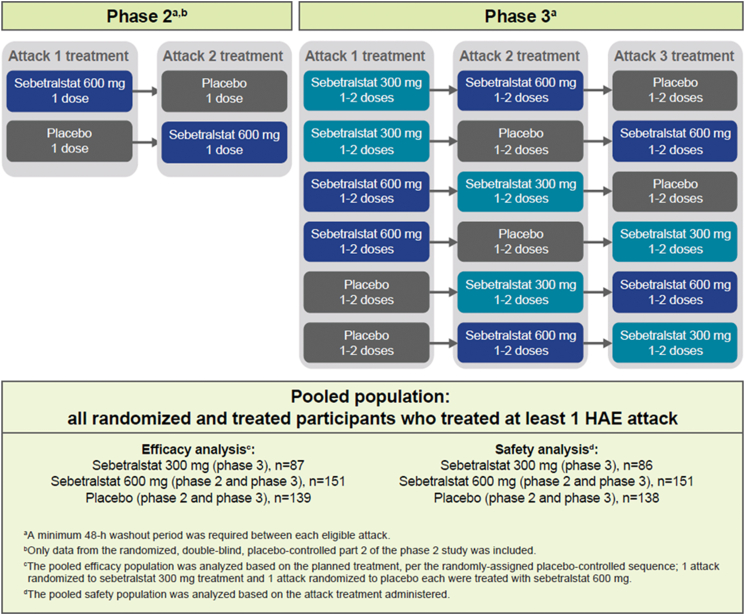
**Trial designs.** HAE. hereditary angioedema

The phase 2 and phase 3 trials of sebetralstat were randomized, double-blind, placebo-controlled, crossover designs and have been previously described (Table 1).^12^^,^^13^ The pooled population included all randomized participants who treated ≥1 qualifying HAE attack with ≥1 dose of sebetralstat or placebo during the double-blind treatment period of each trial (Fig. 1). All participants had access and the ability to self-administer conventional on-demand therapy (plasma-derived C1INH, recombinant human C1INH, icatibant, or ecallantide [phase 3 only]). The efficacy population was analyzed based on the planned treatment, and the safety population was analyzed based on the received treatment.

**Table 1:** Designs of pooled phase 2 and phase 3 sebetralstat trials.^12^^,^^13^

Study	Age	Diagnosis	Documented attacks in the previous 3 months	Prophylactic therapy	Assessment schedule	Number of administrations	Attack location	Attack severity	Sites	Countries
**Phase 2**	≥18 y	HAE-C1INH-Type1 or HAE-C1INH-Type2	≥3	Not allowed	Every 30 min for the first 4 h after first taking the study drug, then every hour from 4 to 12 h, every 3 h from 12 to 24 h, and once at 36 and 48 h	Single	Below the neck	Less than “severe” on the patient global impression–severity scale at baseline	25	10 (regions included Europe and North America)
**Phase 3**	≥12 y	HAE-C1INH-Type1 or HAE-C1INH-Type2	≥2	Stable dose/regimen for at least 3 months before screening	Every 30 min for the first 4 h after first taking the study drug, then every hour from 4 to 12 h, every 2 h from 12 to 24 h, and once at 36 and 48 h	Second optional	Any	Excluded severe laryngeal attacks at baseline (as categorized by the participant)	53	17 (regions included Europe, North America, and Asia-Pacific)

C1INH, C1 inhibitor; h, hours; HAE, hereditary angioedema; HAE-C1INH-Type1, HAE due to C1INH deficiency; HAE-C1INH-Type2; HAE due to C1INH dysfunction.

The trials were approved by the relevant institutional review board or ethics committee, followed good clinical practice guidelines, observed the Declaration of Helsinki, and followed the guidance of the International Conference on Harmonization. All participants provided written informed consent, with a parent or legally authorized representative providing signed informed consent when required.

Participants were randomly assigned to administer a single oral administration of sebetralstat 600 mg or placebo in 1 of 2 sequences (phase 2, 1:1); or sebetralstat 300 mg, sebetralstat 600 mg, or placebo in 1 of 6 predetermined sequences (phase 3, 1:1:1:1:1:1; Fig. 1). Randomization was performed in both trials using a permutated-block method; phase 3 was stratified by use of long-term prophylaxis at enrollment. Both trials were performed in a double-blind manner.

### Attack treatment and characteristics

In the phase 2 trial, attack eligibility was determined by the trial physician (or qualified designee) via telephone with the participant. Participants were instructed to treat attacks within 1 h of recognition. Attacks rated “Severe” or above on the Patient Global Impression−Severity (PGI-S) scale and/or with an attack location of the neck and above were ineligible. Participants could take conventional treatment 4 h after (or earlier, if warranted for severity) study drug administration. Attacks requiring conventional injectable treatment were classified as treatment failures and right censored to the end of the analysis window. A minimum 48-h washout period (of study drug and/or conventional treatment) was required between eligible attacks.

In the phase 3 trial, attack eligibility was determined by the participant; participants were required to be able to identify the start of eligible attacks. Participants were instructed to administer treatment “as early as possible” after attack recognition. Any location was eligible, but due to the possibility of treating potentially life-threatening attacks with placebo, severe laryngeal attacks were not eligible (“Mild”/“Moderate” laryngeal attacks were eligible). An additional dose of the study drug could be taken ≥3 h after the first dose if the participant considered symptoms severe enough to require an additional dose. Conventional on-demand treatment could be taken ≥1 h after the second administration of study drug if attack symptoms were considered severe enough to warrant its use (as determined by the participant). A minimum 48-h washout period (of study drug and/or conventional treatment) was required between eligible attacks.

In both trials, participants were asked to use a diary to record the date and time(s) that the dose(s) of study drug was taken, time from attack onset, location, severity, and use of conventional on-demand treatment, as applicable. Participants recorded the onset of symptom relief and attack severity using the 7-point Patient Global Impression–Change (PGI-C; ratings range from “Much Better” to “Much Worse”) scale and the 5-point PGI-S (ratings range from “None” to “Very Severe”) scale, respectively. Attacks rated by the patient as baseline severity of “None” on the PGI-S were analyzed in the “Mild” group. Assessments were recorded every 30 min for the first 4 h after taking the study drug, every hour from 4 to 12 h, every 3 h (phase 2) or 2 h (phase 3) from 12 to 24 h, at 36 h, and at 48 h.

### Efficacy analysis

Sebetralstat efficacy assessments included time to beginning of symptom relief and reduction in attack severity (both within 12 h), complete attack resolution within 24 h, and use of conventional HAE treatment within 12 h of study drug administration. The beginning of symptom relief was defined as an attack rating of at least “A Little Better” on PGI-C for at least 2 consecutive time points within 12 h of receiving study drug. This endpoint was also evaluated in subgroups: primary attack location (ie, mucosal, abdominal only, subcutaneous); baseline attack severity (ie, “Mild”, “Moderate”, “Severe” or “Very Severe”); age (ie, ≥12 to <18 years, ≥18 years); sex; geographic region (ie, Europe, North America, Asia-Pacific); and baseline treatment regimen (ie, on-demand only, on-demand plus long-term prophylaxis); all subgroup analyses are descriptive and not powered for formal interaction testing. A reduction in attack severity was defined as an improved rating on PGI-S for at least 2 consecutive time points within 12 h of study drug administration. Complete attack resolution was defined as a PGI-S rating of “None” (no symptoms) within 24 h of study drug administration. Additional assessments included time to substantial reduction of symptom burden within 12 h, defined as time to a decrease in PGI-S rating to “Mild” for 2 consecutive time points within 12 h for attacks that had reached the rating of “Moderate” or worse at baseline.

### Safety and tolerability

Safety-related assessments were primarily measured by adverse events (AEs), including seriousness, severity, and association with study drug. Safety data were reported as number and proportion of participants reporting AEs and number of AEs, categorized according to Medical Dictionary for Regulatory Activities, version 26.0, system organ class, and preferred term. To remove the confounding effect of concurrent abdominal attacks on relevant gastrointestinal treatment-emergent AEs (TEAEs) (including preferred terms of abdominal pain, nausea, vomiting, dyspepsia, and diarrhea), the safety population was evaluated to assess the risk difference between relevant gastrointestinal TEAEs occurring with sebetralstat versus placebo among attacks involving subcutaneous or larynx/throat tissue only.

### Statistical analysis

Kaplan-Meier estimates were calculated for median (IQR) time to beginning of symptom relief, reduction in attack severity, complete attack resolution, and substantial reduction of symptom burden. Hazard ratios between treatments for time to beginning of symptom relief were calculated with a proportional hazard model controlling for sequence and period. Attacks treated with conventional HAE attack treatment before the occurrence of the endpoint event were considered as treatment failure and right-censored at the end of analysis window. *P* values were calculated using the Gehan score transformation test and not adjusted for multiplicity and were considered nominal and exploratory. Risk difference was assessed for relevant gastrointestinal-related TEAEs using Statistical Analysis System Programming (version 9.4; SAS Institute for Advanced Analytics, Cary, NC, USA).

## Results

### Participants

Between July 2, 2019, and December 8, 2020, 84 adults (≥18 years) with HAE were screened for the phase 2 trial, 68 were enrolled and randomly assigned to 1 of 2 placebo-controlled sequences. Between February 23, 2022, and July 30, 2023, 158 adolescents (≥12 to <18 years) and adults (≥18 years) with HAE were screened for the phase 3 trial; 136 were enrolled and randomly assigned to 1 of 6 placebo-controlled sequences. Among participants in both trials, 68 in phase 2 and 110 in phase 3 received ≥1 dose of study drug, and 113 (phase 2) and 264 (phase 3) HAE attacks were treated. Participant characteristics were balanced across treatments (Table 2). The number of participants aged ≥12 to <18 years was 13 (7.6%). In the phase 3 trial, 1 attack randomized to sebetralstat 300 mg and 1 attack randomized to placebo each received sebetralstat 600 mg; these attacks were analyzed by their planned treatment in the efficacy analysis and their actual treatment in the safety analysis.

**Table 2:** Pooled characteristics of participants treated with sebetralstat or placebo.

Participant parameter	HAE attacks treated with sebetralstat		HAE attacks treated with placebo (n = 139)
	300 mg (n = 87)	600 mg (n = 151)	
**Age, mean (SD) years**	37.2 (14.7)	38.0 (14.1)	38.5 (14.5)
**Age group, n (%)**			
≥12 to <18 years	10 (11.5)	11 (7.3)	9 (6.5)
≥18 years	77 (88.5)	140 (92.7)	130 (93.5)
**Sex, n (%)**			
Male	33 (37.9)	65 (43.0)	57 (41.0)
Female	54 (62.1)	86 (57.0)	82 (59.0)
**Race, n (%)**			
White	73 (83.9)	138 (91.4)	128 (92.1)
Black	1 (1.1)	0	0
Asian	9 (10.3)	8 (5.3)	7 (5.0)
Other or not reported	4 (4.6)	5 (3.3)	4 (2.9)
**BMI, mean (SD) kg/m^2^**	27.4 (6.4)	27.1 (5.5)	27.1 (5.4)
**Geographic region, n (%)**			
Europe	44 (50.6)	101 (66.9)	98 (70.5)
North America	27 (31.0)	34 (22.5)	28 (20.1)
Asia-Pacific (Australia, Israel, Japan)	16 (18.4)	16 (10.6)	13 (9.4)
**Time since diagnosis,**^a^**years**			
Mean (SD)	14.8 (10.3)	17.1 (12.0)	17.7 (12.3)
Median (IQR)	11.1 (6.2–22.3)	15.0 (7.3–24.0)	17.0 (9.0–24.0)
**Current treatment regimen, n (%)**			
On-demand only	68 (78.2)	130 (86.1)	121 (87.1)
On-demand plus prophylaxis	19 (21.8)	21 (13.9)	18 (12.9)

Participants, based on treatment assigned during crossover, may be represented in multiple columns.

BMI, body mass index; HAE, hereditary angioedema; IQR, interquartile range; SD, standard deviation.

a Date of randomization – date of HAE diagnosis.

### HAE attack characteristics

Of 377 HAE attacks, 87 were treated with sebetralstat 300 mg (which was only included in the phase 3 trial), 151 with sebetralstat 600 mg, and 139 with placebo (Table 3). Attack severity at the time of treatment did not substantially differ among treatments; most were rated as “Mild” (174 [46.2%]) or “Moderate” (153 [40.6%]); baseline PGI-S rating and baseline attack location were missing for 2 attacks treated with sebetralstat 300 mg. At the time of attack onset, 156 attacks (41.4%) were mucosal; of these, 115 (30.5%) were abdominal-only attacks, and 8 (2.1%) were laryngeal attacks.

**Table 3:** HAE attack characteristics.

	Sebetralstat		Placebo (n = 139)	All HAE attacks (N = 377)
	300 mg (n = 87)	600 mg (n = 151)		
**Primary pooled attack location, n (%)**^a^				
Mucosal	36 (41.4)	62 (41.1)	58 (41.7)	156 (41.4)
Laryngeal	2 (2.3)	2 (1.3)	4 (2.9)	8 (2.1)
Abdominal only	24 (27.6)	48 (31.8)	43 (30.9)	115 (30.5)
Abdominal and subcutaneous	10 (11.5)	12 (7.9)	11 (7.9)	33 (8.8)
Subcutaneous	49 (56.3)	89 (58.9)	81 (58.3)	219 (58.1)
**Baseline PGI-S category, n (%)**^a^				
Mild^b^	36 (41.4)	69 (45.7)	69 (49.6)	174 (46.2)
Moderate	35 (40.2)	62 (41.1)	56 (40.3)	153 (40.6)
Severe/Very severe	14 (16.1)	20 (13.2)	14 (10.1)	48 (12.7)
**Time from onset of attack to first administration, median (IQR), minutes**	35.0 (6–130)	32.0 (7–85)	32.0 (13–82)	32.5 (8–94)
**Attacks treated in <60 min, n (%)**	53 (60.9)	103 (68.2)	93 (66.9)	249 (66.0)

Time from onset of attack to first administration was missing for 1 attack treated with sebetralstat 300 mg.

HAE, hereditary angioedema; IQR, interquartile range; PGI-S, Patient Global Impression−Severity.

a Baseline PGI-S rating and baseline attack location are missing for 2 attacks in the sebetralstat 300 mg group.

b Included 2 attacks in the sebetralstat 600 mg group and 2 attacks in the placebo group with a baseline PGI-S score of ‘None’.

Of 377 HAE attacks, 249 (66.0%) were treated <1 h after recognition. Median (IQR) time from attack onset to treatment administration was 32.5 min (8–94) (Supplementary Fig. 1). Attacks treated within the first quartile (≤8 min) were considered to be treated “earlier,” and those within the last quartile (≥94 min) were considered “later.” For sebetralstat 300 mg, sebetralstat 600 mg, and placebo, earlier-treated attacks (43.5%, 55.6%, and 48.0%, respectively) were more likely to be mild compared with later-treated attacks (29.6%, 33.3%, and 31.3%, respectively) (Supplementary Table 1).

### Efficacy

**Fig. 2:**
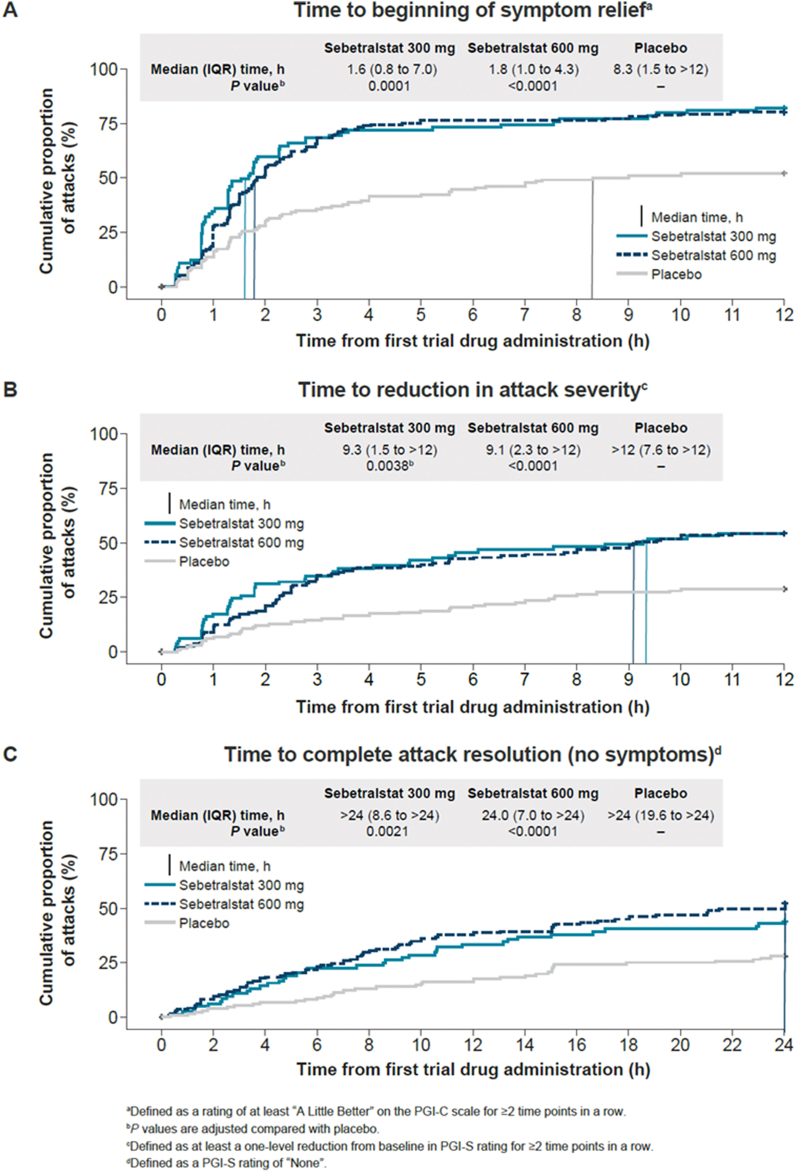
**Time to A) beginning of symptom relief, B) reduction in severity, and C) resolution of HAE attacks.** Subgroup analyses are exploratory in nature and not adjusted for multiplicity. CI, confidence interval; HAE, hereditary angioedema; IQR, interquartile range; NE, not estimable; PGI-C, Patient Global Impression−Change; PGI-S, Patient Global Impression−Severity

Sebetralstat was effective in treating HAE attacks versus placebo (Fig. 2). Compared with placebo, time to beginning of symptom relief was significantly faster with sebetralstat 300 mg (*P* = 0.0001) and 600 mg (*P* < 0.0001), as was reduction in severity (*P* = 0.0038; *P* < 0.0001) and complete attack resolution (*P* = 0.0021; *P* < 0.0001). Higher proportions of attacks treated with sebetralstat 300 mg and 600 mg achieved time to beginning of symptom relief within 12 h versus placebo (66/87 [75.9%], 116/151 [76.8%] vs 69/139 [49.6%]). A higher proportion of attacks treated with sebetralstat 300 mg and 600 mg also achieved reduction in severity within 12 h versus placebo (44/87 [50.6%], 79/151 [52.3%] vs 38/139 [27.3%]). More attacks treated with sebetralstat 300 mg and 600 mg achieved complete resolution within 24 h versus placebo (37/87 [42.5%], 77/151 [51.0%] vs 38/139 [27.3%]).

For HAE attacks rated as “Moderate” or worse at baseline, the median time to substantial reduction of symptom burden within 12 h (IQR) was 5.0 h (1.7 to >12) and 4.6 h (2.2 to >12) for attacks treated with sebetralstat 300 mg and 600 mg, respectively, and was not reached for attacks treated with placebo (Supplementary Table 2). A higher proportion of attacks treated with sebetralstat 300 mg and 600 mg achieved substantial reduction of symptom burden within 12 h versus placebo (34/49 [69.4%], 51/82 [62.2%] vs 26/70 [37.1%]) (Supplementary Fig. 2). Time to attack resolution within 24 h was comparable across both studies, suggesting that the difference in instruction phrasing (phase 2: “within 1 h of recognition” vs phase 3: “as early as possible”^12^^,^^13^) did not significantly affect clinical outcomes.

**Fig. 3:**
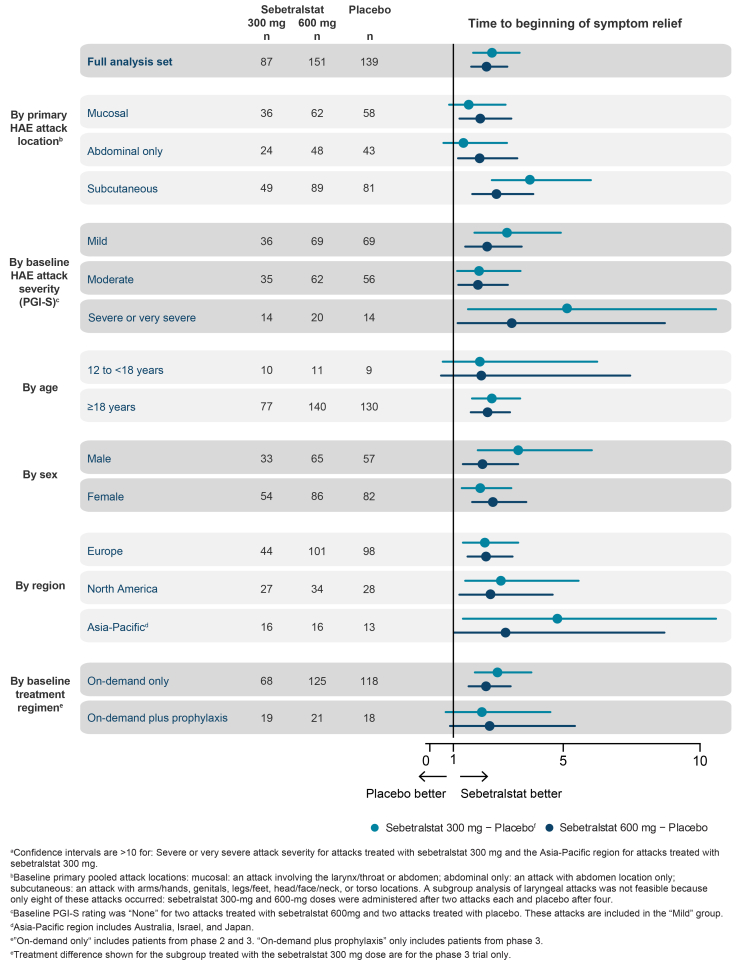
**Efficacy in subgroups, treatment difference (hazard ratios with 95% CI).^a^** Subgroup analyses are exploratory in nature and not adjusted for multiplicity. Hazard ratios were estimated using a standard proportional hazards model. Due to model convergence instability when incorporating shared frailty terms for patient ID, within-participant correlation was not explicitly modeled in the hazard ratio calculation; therefore, confidence intervals should be interpreted with caution. CI, confidence interval; HAE, hereditary angioedema; PGI-S, Patient Global Impression−Severity

In the subgroup analysis, results favoring sebetralstat versus placebo were generally consistent when analyzed by pooled primary HAE attack location (mucosal, abdominal only, or subcutaneous), baseline attack severity, age, sex, geographic region, and treatment regimen (Fig. 3). Of note, HAE attacks with a baseline severity of “Severe” or “Very Severe” more strongly favored sebetralstat over placebo in terms of time to beginning of symptom relief compared with attacks with a baseline severity of “Mild” or “Moderate”, although results should be interpreted with caution due to the wide confidence intervals.

Conventional HAE treatment within 12 h of first study drug administration was used for 12 of 87 attacks (13.8%) treated with sebetralstat 300 mg, 16 of 151 attacks (10.6%) treated with sebetralstat 600 mg, and 38 of 139 attacks (27.3%) treated with placebo. For HAE attacks rated “Severe” or “Very Severe” at baseline, conventional medication was used for 2 of 14 attacks (14.3%) treated with sebetralstat 300 mg, 3 of 20 attacks (15.0%) treated with sebetralstat 600 mg, and 6 of 14 attacks (42.9%) treated with placebo.

### Safety

The safety population was analyzed based on the treatment participants received. An overall similar safety profile was observed between both sebetralstat doses and placebo (Table 4). TEAEs occurred in 17 of 86 participants (19.8%) who received sebetralstat 300 mg, 28 of 151 participants (18.5%) who received sebetralstat 600 mg, and 24 of 138 participants (17.4%) who received placebo. Three serious TEAEs were reported, none of which were considered treatment-related by investigators. Two serious events were reported with sebetralstat 600 mg: 1 event of anisocoria related to lisdexamfetamine use and 1 event of exacerbation of an attack, for which the participant did not take the study drug. The only severe TEAE, as determined by investigator, was 1 event of lumbar disk herniation requiring hospitalization that occurred with sebetralstat 300 mg.

**Table 4:** TEAEs.

TEAE, number of participants (%), E	Sebetralstat		Placebo (n = 138)
	300 mg (n = 86)	600 mg (n = 151)	
**Any TEAE**	**17 (19.8) 20**	**28 (18.5) 39**	**24 (17.4) 34**
Treatment-related TEAE	2 (2.3) 2	6 (4.0) 7	6 (4.3) 7
**Any serious TEAE**	**1 (1.2) 1**	**2 (1.3) 2**	**0**
Treatment-related serious TEAE	0	0	0
**Any severe TEAE**	**1 (1.2) 1**	**0**	**0**
Treatment-related severe TEAE	0	0	0
**Any on-treatment TEAE**	5 (5.8) 5	14 (9.3) 18	16 (11.6) 21
**Treatment-related on-treatment TEAE**	2 (2.3) 2	5 (3.3) 5	6 (4.3) 7
**Any TEAE leading to study discontinuation**	0	0	0
**Any TEAE leading to death**	0	0	0

Bold is used to identify categories.

Abbreviations: E, number of events; TEAE, treatment-emergent adverse event.

Treatment-related TEAEs occurred in 2 of 86 participants (2.3%) who received sebetralstat 300 mg, 6 of 151 participants (4.0%) who received sebetralstat 600 mg, and 6 of 138 participants (4.3%) who received placebo (Table 5). TEAEs that occurred while on treatment (within 3 days of study drug administration) were similar across treatments (Supplementary Table 3). TEAEs were also similar when separated by dose level (Supplementary Tables 4 and 5) and attack location (Supplementary Table 6). No occurrences of dysphagia or TEAEs related to swallowing were reported. No deaths or discontinuations due to AEs occurred.

**Table 5:** Treatment-related TEAEs.

System organ class, preferred term, n (%), E	Sebetralstat		Placebo (n = 138)
	300 mg (n = 86)	600 mg (n = 151)	
**Treatment-related TEAE**	**2 (2.3) 2**	**6 (4.0) 7**	**6 (4.3) 7**
**Gastrointestinal disorders**	**1 (1.2) 1**	**3 (2.0) 3**	**2 (1.4) 2**
Dyspepsia	1 (1.2) 1	1 (0.7) 1	0
Upper abdominal pain	0	1 (0.7) 1	0
Nausea	0	1 (0.7) 1	1 (0.7) 1
Anal incontinence	0	0	1 (0.7) 1
**General disorders and administration site conditions**	**1 (1.2) 1**	**0**	**0**
Fatigue	1 (1.2) 1	0	0
**Musculoskeletal and connective tissue disorders**	**0**	**1 (0.7) 1**	**0**
Back pain	0	1 (0.7) 1	0
**Nervous system disorders**	**0**	**2 (1.3) 2**	**3 (2.2) 3**
Headache	0	2 (1.3) 2	2 (1.4) 2
Dysgeusia	0	0	1 (0.7) 1
**Vascular disorders**	**0**	**1 (0.7) 1**	**0**
Hot flush	0	1 (0.7) 1	0
**Reproductive system and breast disorders**	**0**	**0**	**1 (0.7) 1**
Irregular menstruation	0	0	1 (0.7) 1
**Skin and subcutaneous tissue disorders**	**0**	**0**	**1 (0.7) 1**
Rash	0	0	1 (0.7) 1

Bold is used to identify categories.

E, number of events; n, number of participants with at least 1 adverse event; TEAE, treatment-emergent adverse event.

Risk difference analysis in the safety population identified relevant gastrointestinal TEAEs with a preceding HAE attack not involving the abdomen. In such attacks, there were no gastrointestinal TEAEs reported, with preceding attacks treated with sebetralstat 300 mg; 3 patients (3.3% among attacks treated with sebetralstat 600 mg, 2.7% among all sebetralstat-treated attacks) had 4 gastrointestinal TEAEs with preceding attacks treated with sebetralstat 600 mg, and 3 patients (3.6%) had 5 gastrointestinal TEAEs with preceding attacks treated with placebo. The risk difference (95% CI) was −0.8 (−5.9-4.3) for the pooled sebetralstat doses versus placebo, implying no difference in risk of gastrointestinal TEAEs occurring with sebetralstat versus placebo.

## Discussion

Sebetralstat was developed as an oral alternative to injectable on-demand HAE therapies, which have proven difficult for patients to administer and have been associated with treatment delays and injection-site reactions.^6^^,^^10^^,^^19^, ^20^, ^21^, ^22^, ^23^ In this study, the largest pooled placebo-controlled analysis of HAE on-demand treatment to date, consistent results across the phase 2 and 3 sebetralstat trials demonstrated reproducibility and offered evidence of efficacy for treating attacks among adolescents and adults with HAE, though any statistics are nominal and should be considered exploratory.

Unlike trials of parenteral on-demand HAE treatments, the phase 2 and 3 trials of sebetralstat were designed with the goal of treating attacks early, aiming to intervene before symptomatic progression. Among 377 attacks treated with study drug, median time from attack recognition to treatment was 32.5 min, with 66% of attacks treated within <1 h. Most prior trials required patients to travel to a study site for treatment administration by a healthcare professional within 4–12 h after attack onset.^5^ Although not reported consistently in prior on-demand HAE trials, time from onset of symptoms to administration of treatment was substantially longer for parenterally administered on-demand treatments versus sebetralstat.^24^^,^^25^ Pooled results of randomized clinical trials of recombinant human C1INH had a median time from attack onset to start of a treatment infusion of 240 (with 100 U/kg) or 347 (with 50 U/kg) minutes.^24^ In the phase 3 FAST-3 trial of icatibant, median time from attack onset to treatment was 6.5 h.^25^

Consistent with the concept that earlier use of on-demand HAE therapy can reduce attack severity, 46.2% of attacks were mild at the time of treatment. The baseline severity of attacks that were treated within the shortest time-to-treatment quartile was more frequently mild when compared with attacks treated in the longest time-to-treatment quartile.

Direct comparisons between clinical trials of on-demand HAE treatments are complicated by differences in their study designs.^5^ In contrast to the trials of sebetralstat, many earlier trials of parenteral treatments limited eligible attacks to those rated moderate or severe, resulting in an unknown number of untreated mild attacks that did not progress.^5^ In addition to different requirements for time to treatment and baseline severity, different efficacy endpoints were used across the pivotal trials for the 4 parenteral on-demand treatments approved before sebetralstat, and different metrics were used to assess symptom relief.^5^^,^^26^

The similar designs of the sebetralstat phase 2 and 3 trials allowed results to be pooled, providing more precise estimates of efficacy and additional participant numbers for subgroup analyses. This pooled analysis showed efficacy across dose, anatomic location, attack severity, age, sex, geographic region, and baseline treatment regimen. Subgroup analysis was previously limited in the individual trials by low numbers of attacks, highlighting the unique advantage of a similar trial design in facilitating pooled post hoc analyses by providing more attacks per subgroup to demonstrate the consistency and robustness of the results, though in the absence of formal interaction testing, results should be considered descriptive and hypothesis-generating.

In this pooled analysis, sebetralstat was well tolerated, with a safety profile no different from that of the placebo.^12^^,^^13^ No treatment-related TEAEs were serious, severe, or resulted in death or discontinuation. No occurrences of dysphagia or TEAEs related to swallowing were reported. The incidence of vomiting was low (1 event with sebetralstat 300 mg, 2 events with sebetralstat 600 mg, 3 events with placebo), which is an important consideration for oral therapies. Although it can be challenging to distinguish gastrointestinal TEAEs from abdominal HAE attacks with similar symptoms, a risk difference analysis excluding nonabdominal attacks removed this confounding factor and showed a comparable risk of gastrointestinal TEAEs between sebetralstat and placebo.

### Limitations

Combining data from different studies often requires careful consideration of potential differences that can introduce heterogeneity, potentially leading to biased pooled estimates. There were several differences between the phase 2 and 3 sebetralstat trials: a 2-way crossover design in phase 2 versus a 3-way crossover design in phase 3; differences in study populations (ie, permitted long-term prophylaxis; inclusion of adolescent patients); and the option for a second administration of sebetralstat in phase 3. Finally, the phase 2 and 3 trials utilized different primary efficacy endpoints. While the endpoints analyzed were defined the same way across both studies, potentially mitigating this difference, this could influence the robustness and generalizability of the pooled results, and data should be interpreted with caution. Although it has been argued that a limitation of the phase 2 sebetralstat trial was lack of data for abdominal attacks,^27^ approximately 40% of attacks in this pooled analysis involved the abdomen, and sebetralstat showed superior efficacy over placebo in this subgroup. Because of trial design, there were no laryngeal attacks in phase 2, and too few occurred in the phase 3 trial to draw conclusions related to treatment of laryngeal attacks. Laryngeal attacks of any severity are being evaluated in the ongoing, open-label KONFIDENT-S study (NCT05505916, EudraCT: 2021-001176-42) with reported interim analyses.^28^

### Conclusion

This pooled analysis of results from the phase 2 and 3 trials of sebetralstat in participants with HAE confirmed the initial trial findings, with an increased sample size, while permitting a deeper exploration of safety and efficacy in subgroups. Both trials of sebetralstat were unique in that they prioritized early treatment and did not require participants to meet a minimum baseline attack severity before treatment, in line with current guidelines for treatment of HAE attacks.^1^^,^^2^ Pooled results show that sebetralstat can offer improved outcomes across doses and subgroups. Attacks treated earlier were associated with milder attack severity, supporting the recommendation for treatment as soon as possible after recognition. As the first oral on-demand treatment, sebetralstat has the potential to transform the management of HAE attacks. Its convenient oral administration route may overcome adherence barriers associated with injectable therapies, enabling earlier intervention and improving patients’ quality of life.

## Availability of data and materials

KalVista accepts requests from qualified researchers who wish to access clinical trial data and associated information, such as Clinical Study Reports (CSRs) with appropriately redacted appendices to protect participant privacy. Please direct your inquiry to DSP@kalvista.com for more details.

## Author contributions

MDS, JH, MI, CMY, PKA contributed to conception and design of the study. EA-P, DMC, SK-A, and MM served as investigators on the clinical trials and verified the underlying study data. JH contributed to the data analysis. All authors contributed to the interpretation of the results. All authors critically reviewed and revised the manuscript for intellectual content. All authors read and approved the final manuscript. All authors agree to be accountable for the accuracy and integrity of the work.

## Ethics statement

The trials were approved by the relevant institutional review board or ethics committee, followed good clinical practice guidelines, observed the Declaration of Helsinki, and followed the guidance of the International Conference on Harmonization. All participants provided written informed consent, with a parent or legally authorized representative providing signed informed consent when required. Clinical trial registration: ClinicalTrials.gov Identifier NCT04208412, registered on 2019-07-02; ClinicalTrials.gov Identifier NCT05259917 (KONFIDENT), registered on 2022-02-22.

## Declaration of generative AI and AI-assisted technologies in the writing process

Nothing to disclose.

## Funding

The individual trials and the pooled analysis were sponsored by KalVista Pharmaceuticals, Inc. Medical writing support was provided by Marisa DeGuzman, PhD, and Heather A. Mitchell, PhD, of Oxford PharmaGenesis Inc., Wilmington, DE, USA, and funded by KalVista Pharmaceuticals, Inc.

**Table:** 

Author	Disclosure
Emel Aygören-Pürsün	Has received grants, consulting fees, honoraria, fees paid to the institution, and/or personal fees from KalVista pharmaceuticals, Astria, BioCryst, Centogene, CSL Behring, Intellia, Otsuka, Pharvaris, and Takeda/Shire.
Nancy Agmon-Levin	The author reports no competing interests.
Paul K. Audhya	Is an employee of KalVista pharmaceuticals.
Aleena Banerji	Has received research grants from Takeda and BioCryst and consulting fees from Takeda, BioCryst, pharming, CSL, Pharvaris, KalVista, and Biomarin.
Jonathan A. Bernstein	Has received grants and/or honoraria from KalVista pharmaceuticals, BioCryst, BioMarin, CSL Behring, Intellia, Ionis, pharming, Pharvaris, and Takeda/Shire and serves as the immediate past president of the American academy of allergy, asthma & immunology (AAAAI).
Paula Busse	Has received consulting fees and/or research grants/contracts from ADARx, Amgen Inc., Astria, BioCryst, BioMarin, CSL Behring, CVS specialty, Intellia, KalVista, Novartis, Pharvaris, Regeneron, Sanofi Pasteur Biologics LLC, and Takeda; has served as expert witness for Hinckley Allen; and has served as medical advisor for the hereditary angioedema association (HAEA).
Mauro Cancian	Has received funding for research projects from or served on advisory boards for CSL Behring, BioCryst, and Takeda/Shire.
Eunice Dias de Castro	Has received speaker honoraria, meeting/travel support, and/or served on advisory boards for CSL Behring, BioCryst, KalVista, and Takeda.
Danny M. Cohn	Has received consulting fees paid to the institution, honoraria paid to the institution, meeting/travel support, and research support; has served on advisory boards for KalVista, Astria, BioCryst, CSL Behring, Intellia, Ionis pharmaceuticals, Intellia, Otsuka, Pharvaris, and Takeda; and has a leadership role for the HAE International (HAEi) Medical Advisory panel for Central Eastern Europe and Benelux.
Henriette Farkas	Has received grants paid to the institution, honoraria, meeting/travel support, and/or served on advisory boards for KalVista, Astria, BioCryst, CSL Behring, Intellia, Ono pharmaceutical, pharming, Pharvaris, and Takeda and has served a leadership role on the angioedema centers of reference and excellence (ACARE) steering committee.
Vesna Grivcheva-Panovska	Has served on advisory boards for CSL Behring, Takeda, BioCryst, KalVista, and Pharvaris; has received honoraria for presentations from CSL Behring and Takeda, an unrestricted research grant from CSL Behring, and travel assistance from CSL Behring.
David Hagin	Has received consultant fees from KalVista.
Roman Hakl	Has received consultancy or speaker honoraria from CSL Behring, Shire/Takeda, and has served as a principal investigator for clinical trials sponsored by BioCryst, Pharvaris Netherlands BV, pharming, CSL Behring, and KalVista.
James Hao	Is an employee of KalVista pharmaceuticals.
Daisuke Honda	Has received consultant and/or speaker/advisor fees from BioCryst, CSL Behring, diagnostic consortium to advance the ecosystem for hereditary angioedema (DISCOVERY), KalVista, Takeda, and Torii; has served as cooperating physician for HAE Japan (HAEJ).
Matthew Iverson	Is an employee of KalVista pharmaceuticals.
Aharon Kessel	Has received speaker/advisor fees and/or travel support from KalVista and Takeda.
Sorena Kiani-Alikhan	Has received speaker or consultancy fees and grants from BioCryst, CSL Behring, pharming, Takeda, KalVista, Ionis, Astria and Otsuka UK.
Tamar Kinaciyan	Is or recently was a speaker and/or advisor for and/or has received research funding from: BioCryst, CSL Behring, KalVista, Otsuka, Pharvaris, Sanofi-Aventis, and Takeda.
Marcin Kurowski	Has received speaker honoraria and/or travel support from CSL Behring, pharming healthcare, Inc., and Takeda; has served as an advisory board member for KalVista.
H. Henry Li	Has received speaker or consultant fees from KalVista, BioCryst, CSL Behring, Ionis, pharming, and Takeda. Participated in sponsored clinical studies by KalVista, CSL Behring, BioCryst, pharming, Pharvaris, Ionis, and Intellia
Ramon Lleonart	Has received consulting fees, honoraria, payment for expert testimony, meeting/travel support, and/or served on advisory boards for KalVista, BioCryst, CSL Behring, pharming, and Takeda.
Hilary J. Longhurst	Has served as consultant, speaker, or engaged in research with or educational projects with Astria, CSL Behring, Intellia, KalVista, Pharvaris, and Takeda.
William R. Lumry	Has served as a consultant, speaker or engaged in research or educational projects with Astria, Astra Zeneca, BioCryst, Biomarin, CSL Behring, Express scripts/CVS, Grifols, Intellia, KalVista, Eli Lilly, Magellan, Novartis, Optum, pharming, Pharvaris, Shire/Takeda and upstream bio. and is a medical advisory board member of the US hereditary angioedema association.
Markus Magerl	Received personal fees/nonfinancial support from Astria, Shire Takeda, CSL Behring, pharming, BioCryst, KalVista, Pharvaris, Ionis, Intellia, and Octapharma.
Inmaculada Martinez-Saguer	Has received speaker or consultancy fees from BioCryst, CSL Behring, Pharming, and Takeda, and/or served on advisory boards for BioCryst, CSL Behring, KalVista, Octapharma, Pharming, Pharvaris, and Takeda.
Isaac Melamed	Has no disclosures.
Samuel Owiredu-Yeboa Ketan Patil	Is an employee of KalVista Pharmaceuticals. Is an employee of KalVista Pharmaceuticals.
Fotis Psarros	Has received speaker honoraria and/or served on a scientific advisory board for CSL Behring, Pharvaris GmBH, and Takeda; has been principal investigator for KalVista and Takeda clinical trials.
Marc A. Riedl	Is or recently was a speaker and/or advisor for and/or has received research funding from Astria, BioCryst, BioMarin, Celldex, CSL Behring, Cycle Pharma, Grifols, Intellia, Ionis, KalVista, Novartis, Pharming, Pharvaris, Sanofi-Regeneron, and Takeda.
Sinisa Savic	Has participated in advisory boards and/or received speaker/consultant fees for AstraZeneca UK limited, BioCryst, Celldex Therapeutics, KalVista, Novartis, Pharvaris, Sobi, Inc., and Takeda; has received institutional research grants/contracts from CSL Behring and Novartis.
Michael D. Smith	Is an employee of KalVista pharmaceuticals.
Daniel F. Soteres	Has received speaker/consultant fees, participated in advisory boards, and/or been a primary investigator in clinical research trials for BioCryst, CSL Behring, Ionis, Intellia, KalVista, Pharming, Pharvaris, and Takeda.
Maria Staevska	Has received speaker/consultant fees from CSL Behring, Pharming, and Takeda.
Marcin Stobiecki	Has received speaker/consultant fees KalVista, Pharvaris, Takeda, CSL Behring and Pharming, participated in advisory boards, and/or been a primary investigator in clinical research trials for BioCryst, KalVista, Pharvaris.
H. James Wedner	Has received consultant/speaker fees from BioMarin, CSL Behring, and Takeda; and has received institutional research grants from CSL Behring, Ionis, Pharvaris, and Takeda.
Andrea Zanichelli	Has received honoraria, meeting/travel support, and/or served on advisory boards for KalVista, Astria, BioCryst, CSL Behring, Intellia, Pharming, Pharvaris, and Takeda.
Christopher M. Yea	Is an employee of KalVista pharmaceuticals.
Marcus Maurer	Was a speaker and/or advisor for and/or has received research funding from Astria, BioCryst, CSL Behring, KalVista, Pharvaris, and Takeda.
