# Supplementary material for: Sebetralstat for on-demand treatment of hereditary angioedema: A pooled analysis of placebo-controlled clinical trials
Source: World Allergy Organ J. 2026 Jun 9;19(7):101401. doi: 10.1016/j.waojou.2026.101401 (PMC13273462; doi:10.1016/j.waojou.2026.101401)
Supplement: Multimedia component 1 [file mmc1.docx]

**Supplementary Appendix**

**Supplementary Tables**

| Parameter | Earlier (≤8 min) | | | Later (≥94 min) | | |
| --- | --- | --- | --- | --- | --- | --- |
|  | **Sebetralstat** | | **Placebo**  **(n=25)** | **Sebetralstat** | | **Placebo**  **(n=32)** |
|  | **300 mg**  **(n=23^a^)** | **600 mg**  **(n=36)** |  | **300 mg**  **(n=27)** | **600 mg**  **(n=36)** |  |
| **Baseline PGI-S category, n (%)** | | | | | | |
| Mild | 10 (43.5) | 20 (55.6) | 12 (48.0) | 8 (29.6) | 12 (33.3) | 11 (34.3) |
| Moderate | 7 (30.4) | 10 (27.8) | 10 (40.0) | 14 (51.9) | 18 (50.0) | 15 (46.9) |
| Severe/Very severe | 5 (21.7) | 6 (16.7) | 3 (12.0) | 5 (18.5) | 6 (16.7) | 6 (18.8) |
| **Primary pooled attack location,^b^ n (%)** | | | | | | |
| Laryngeal | 1 (4.3) | 1 (2.8) | 1 (4.0) | 1 (3.7) | 1 (2.8) | 2 (6.3) |
| Abdominal only | 4 (17.4) | 11 (30.6) | 7 (28.0) | 6 (22.2) | 12 (33.3) | 9 (28.1) |
| Subcutaneous only | 13 (56.5) | 23 (63.9) | 15 (60.0) | 16 (59.3) | 20 (55.6) | 18 (56.3) |
| Abdominal and subcutaneous | 4 (17.4) | 1 (2.8) | 2 (8.0) | 4 (14.8) | 3 (8.3) | 3 (9.4) |

**Supplementary Table 1.** Characteristics of treated HAE attacks by time from attack onset to treatment.

Note: Instructions on when to treat differed between the phase 2 and phase 3 trials.

^a^One participant had missing data for both baseline PGI-S category and baseline attack location.

^b^Participants with multiple attack locations are counted once in each reported location.

HAE, hereditary angioedema; PGI-S, Patient Global Impression−Severity.

|  | **Sebetralstat** | | **Placebo**  **(n=70)** |
| --- | --- | --- | --- |
|  | **300 mg**  **(n=49)** | **600 mg**  **(n=82)** |  |
| **Number of patients**  **Events^a^, n (%)** | **34 (69.4)** | **51 (62.2)** | **26 (37.1)** |
| **Time to PGI-S rating of “Mild” for attacks rated “Moderate” or worse at baseline,^b^ h**  Nominal *P* value vs placebo  Median  IQR | 0.0078  5.0  1.7 to >12 | 0.0013  4.6  2.2 to >12 | –  >12  4.0 to >12 |

**Supplementary Table 2.** Time to PGI-S rating of “Mild” for HAE attacks rated “Moderate” or worse at baseline.

^a^”Moderate” or worse attacks at baseline, which achieved PGI-S rating of “Mild” within 12 h.

^b^Kaplan-Meier estimates for time to PGI-S of “Mild” or lower within 12 h.

h, hours; HAE, hereditary angioedema; IQR, interquartile range; PGI-S, Patient Global Impression−Severity.

| **System organ class, preferred term, n (%), E^a^** | **Sebetralstat** | | **Placebo**  **(n=138)** |
| --- | --- | --- | --- |
|  | **300 mg**  **(n=86)** | **600 mg**  **(n=151)** |  |
| **Any TEAE** | **5 (5.8) 5** | **14 (9.3) 18** | **16 (11.6) 21** |
| **Ear and labyrinth disorders**  Vertigo | **0**  0 | **1 (0.7) 1**  1 (0.7) 1 | **0**  0 |
| **Eye disorders**  Eye hemorrhage | **0**  0 | **0**  0 | **1 (0.7) 1**  1 (0.7) 1 |
| **Gastrointestinal disorders**  Dyspepsia  Gingival bleeding  Vomiting  Abdominal pain  Upper abdominal pain  Anal incontinence  Dry mouth  Nausea | **3 (3.5) 3**  1 (1.2) 1  1 (1.2) 1  1 (1.2) 1  0  0  0  0  0 | **5 (3.3) 7**  0  0  2 (1.3) 2  0  1 (0.7) 1  0  1 (0.7) 1  3 (2.0) 3 | **5 (3.6) 7**  0  0  2 (1.4) 3  1 (0.7) 1  1 (0.7) 1  1 (0.7) 1  0  1 (0.7) 1 |
| **General disorders and administration site conditions**  Fatigue | **1 (1.2) 1**  1 (1.2) 1 | **0**  0 | **1 (0.7) 1**  1 (0.7) 1 |
| **Infections and infestations**  Pharyngitis streptococcal  Cystitis  Influenza  Nasopharyngitis  Pharyngitis bacterial | **1 (1.2) 1**  1 (1.2) 1  0  0  0  0 | **1 (0.7) 1**  0  1 (0.7) 1  0  0  0 | **4 (2.9) 4**  0  0  1 (0.7) 1  2 (1.4) 2  1 (0.7) 1 |
| **Musculoskeletal and connective tissue disorders**  Back pain  Neck pain | **0**  0  0 | **1 (0.7) 1**  1 (0.7) 1  0 | **1 (0.7) 1**  0  1 (0.7) 1 |
| **Nervous system disorders**  Dizziness  Dysgeusia  Headache | **0**  0  0  0 | **7 (4.6) 7**  1 (0.7) 1  0  6 (4.0) 6 | **4 (2.9) 4**  0  1 (0.7) 1  3 (2.2) 3 |
| **Reproductive system and breast disorders**  Irregular menstruation | **0**  0 | **0**  0 | **1 (0.7) 1**  1 (0.7) 1 |
| **Respiratory, thoracic, and mediastinal disorders**  Epistaxis | **0**  0 | **0**  0 | **1 (0.7) 1**  1 (0.7) 1 |
| **Skin and subcutaneous tissue disorders**  Rash  Rash erythematous | **0**  0  0 | **1 (0.7) 1**  0  1 (0.7) 1 | **1 (0.7) 1**  1 (0.7) 1  0 |

**Supplementary Table 3.** TEAEs that occurred within 3 days of study treatment.

^a^System organ class and preferred term coded using MedDRA, version 26.0.

E, number of events; MedDRA, Medical Dictionary for Regulatory Activities; n, number of participants with at least 1 adverse event; TEAE, treatment-emergent adverse event.

| **TEAE, number of participants (%), E^a^** | **Sebetralstat** | | | | **Placebo**  **1 or 2 administrations**  **(n=138)** |
| --- | --- | --- | --- | --- | --- |
|  | **300 mg** | | **600 mg** | |  |
|  | **1 administration**  **(n=53)** | **2 administrations**  **(n=33)** | **1**  **administration**  **(n=114)** | **2 administrations**  **(n=38)** |  |
| **Any TEAE** | 9 (17.0) 12 | 8 (24.2) 8 | 20 (17.5) 29 | 8 (21.1) 10 | 24 (17.4) 34 |
| Treatment-related TEAE | 2 (3.8) 2 | 0 | 4 (3.5) 4 | 2 (5.3) 3 | 6 (4.3) 7 |
| On-treatment TEAE^b^ | 4 (7.5) 4 | 1 (3.0) 1 | 11 (9.6) 15 | 3 (7.9) 3 | 16 (11.6) 21 |
| On-treatment treatment-related TEAE^b^ | 2 (3.8) 2 | 0 (0.0) 0 | 4 (3.5) 4 | 1 (2.6) 1 | 6 (4.3) 7 |
| **Any TEAE of grade ≥3** | 1 (1.9) 1 | 0 | 0 | 0 | 0 |
| Any treatment-related TEAE of grade ≥3 | 0 | 0 | 0 | 0 | 0 |
| **Any serious TEAE** | 1 (1.9) 1 | 0 | 2 (1.8) 2 | 0 | 0 |
| Any treatment-related serious TEAE | 0 | 0 | 0 | 0 | 0 |
| **Any TEAE leading to hospitalization** | 1 (1.9) 1 | 0 | 2 (1.8) 2 | 0 | 0 |
| **Any TEAE leading to study discontinuation** | 0 | 0 | 0 | 0 | 0 |
| **Any TEAE leading to death** | 0 | 0 | 0 | 0 | 0 |

**Supplementary Table 4.** Summary of TEAEs by number of sebetralstat administrations.

^a^System organ class and preferred term coded using MedDRA, version 26.0.

^b^On-treatment TEAE was defined as occurring from the first administration of study drug to within 3 days after the last administration of study drug for an attack.

E, number of events; MedDRA, Medical Dictionary for Regulatory Activities; n, number of participants with at least 1 adverse event; TEAE, treatment-emergent adverse event.

| **System organ class, preferred term, n (%), E^a^** | **Sebetralstat** | | | | **Placebo**  **1 or 2 administrations**  **(n=138)** |
| --- | --- | --- | --- | --- | --- |
|  | **300 mg** | | **600 mg** | |  |
|  | **1 administration**  **(n=53)** | **2 administrations**  **(n=33)** | **1 administration**  **(n=114)** | **2 administrations**  **(n=38)** |  |
| **Any TEAE** | 9 (17.0) 12 | 8 (24.2) 8 | 12 (10.5) 29 | 8 (21.1) 10 | 21 (15.2) 34 |
| **Congenital, familial, and genetic disorders** | **0** | **0** | **1 (0.9) 1** | **0** | **0** |
| Hereditary angioedema | 0 | 0 | 1 (0.9) 1 | 0 | 0 |
| **Ear and labyrinth disorders** | **0** | **0** | **1 (0.9) 1** | **0** | **0** |
| Vertigo | 0 | 0 | 1 (0.9) 1 | 0 | 0 |
| **Eye disorders** | **0** | **0** | **1 (0.9) 1** | **0** | **1 (0.7) 1** |
| Anisocoria | 0 | 0 | 1 (0.9) 1 | 0 | 0 |
| Eye hemorrhage | 0 | 0 | 0 | 0 | 1 (0.7) 1 |
| **Gastrointestinal disorders** | **3 (5.7) 3** | **1 (3.0) 1** | **4 (3.5) 6** | **4 (10.5) 4** | **6 (4.3) 8** |
| Abdominal pain | 0 | 0 | 0 | 1 (2.6) 1 | 1 (0.7) 1 |
| Upper abdominal pain | 0 | 0 | 1 (0.9) 1 | 0 | 1 (0.7) 1 |
| Anal incontinence | 0 | 0 | 0 | 0 | 1 (0.7) 1 |
| Dental caries | 1 (1.9) 1 | 0 | 0 | 0 | 0 |
| Dry mouth | 0 | 0 | 1 (0.9) 1 | 0 | 0 |
| Dyspepsia | 1 (1.9) 1 | 0 | 0 | 1 (2.6) 1 | 0 |
| Gingival bleeding | 0 | 1 (3.0) 1 | 0 | 0 | 0 |
| Nausea | 0 | 0 | 2 (1.8) 2 | 1 (2.6) 1 | 1 (0.7) 1 |
| Stomatitis | 0 | 0 | 1 (0.9) 1 | 0 | 0 |
| Toothache | 0 | 0 | 0 | 0 | 1 (0.7) 1 |
| Vomiting | 1 (1.9) 1 | 0 | 1 (0.9) 1 | 1 (2.6) 1 | 2 (1.4) 3 |
| **General disorders and administration site conditions** | **1 (1.9) 1** | **0** | **0** | **0** | **1 (0.7) 1** |
| Fatigue | 1 (1.9) 1 | 0 | 0 | 0 | 1 (0.7) 1 |
| **Immune system disorders** | **0** | **1 (3.0) 1** | **0** | **0** | **0** |
| Seasonal allergy | 0 | 1 (3.0) 1 | 0 | 0 | 0 |
| **Infections and infestations** | **1 (1.9) 1** | **3 (9.1) 3** | **4 (3.5) 5** | **1 (2.6) 1** | **7 (5.1) 7** |
| COVID-19 | 0 | 1 (3.0) 1 | 0 | 1 (2.6) 1 | 0 |
| Cystitis | 0 | 0 | 2 (1.8) 2 | 0 | 0 |
| Fungal skin infection | 0 | 1 (3.0) 1 | 0 | 0 | 0 |
| Influenza | 0 | 0 | 1 (0.9) 1 | 0 | 1 (0.7) 1 |
| Laryngitis | 0 | 1 (3.0) 1 | 0 | 0 | 0 |
| Localized infection | 0 | 0 | 0 | 0 | 1 (0.7) 1 |
| Nasopharyngitis | 0 | 0 | 0 | 0 | 2 (1.4) 2 |
| Oral herpes | 0 | 0 | 1 (0.9) 1 | 0 | 0 |
| Pharyngitis | 0 | 0 | 0 | 0 | 1 (0.7) 1 |
| Pharyngitis bacterial | 0 | 0 | 0 | 0 | 1 (0.7) 1 |
| Pharyngitis streptococcal | 1 (1.9) 1 | 0 | 0 | 0 | 0 |
| Upper respiratory tract infection | 0 | 0 | 1 (0.9) 1 | 0 | 0 |
| Viral upper respiratory tract infection | 0 | 0 | 0 | 0 | 1 (0.7) 1 |
| **Injury, poisoning, and procedural complications** | **0** | **0** | **1 (0.9) 1** | **0** | **1 (0.7) 1** |
| Contusion | 0 | 0 | 0 | 0 | 1 (0.7) 1 |
| Face injury | 0 | 0 | 1 (0.9) 1 | 0 | 0 |
| **Investigations** | **2 (3.8) 5** | **0** | **3 (2.6) 3** | **1 (2.6) 1** | **2 (1.4) 2** |
| Alanine aminotransferase increased | 0 | 0 | 1 (0.9) 1 | 0 | 1 (0.7) 1 |
| Albumin urine present | 1 (1.9) 1 | 0 | 0 | 0 | 0 |
| Blood glucose increased | 0 | 0 | 1 (0.9) 1 | 0 | 0 |
| Blood pressure increased | 0 | 0 | 1 (0.9) 1 | 0 | 0 |
| Blood triglycerides increased | 1 (1.9) 1 | 0 | 0 | 0 | 0 |
| Blood urine present | 1 (1.9) 1 | 0 | 0 | 0 | 0 |
| Gamma-glutamyl transferase increased | 0 | 0 | 0 | 1 (2.6) 1 | 0 |
| Glucose urine present | 1 (1.9) 1 | 0 | 0 | 0 | 0 |
| Mean cell volume increased | 1 (1.9) 1 | 0 | 0 | 0 | 0 |
| Weight decreased | 0 | 0 | 0 | 0 | 1 (0.7) 1 |
| **Metabolism and nutrition disorders** | **0** | **0** | **1 (0.9) 1** | **0** | **0** |
| Hypoglycemia | 0 | 0 | 1 (0.9) 1 | 0 | 0 |
| **Musculoskeletal and connective tissue disorders** | **1 (1.9) 1** | **1 (3.0) 1** | **2 (1.8) 2** | **0** | **1 (0.7) 1** |
| Back pain | 0 | 0 | 1 (0.9) 1 | 0 | 0 |
| Intervertebral disk protrusion | 1 (1.9) 1 | 0 | 0 | 0 | 0 |
| Muscle tightness | 0 | 0 | 1 (0.9) 1 | 0 | 0 |
| Neck pain | 0 | 0 | 0 | 0 | 1 (0.7) 1 |
| Pain in extremity | 0 | 1 (3.0) 1 | 0 | 0 | 0 |
| **Nervous system disorders** | **0** | **1 (3.0) 1** | **6 (5.3) 6** | **2 (5.3) 2** | **6 (4.3) 6** |
| Amnesia | 0 | 0 | 0 | 0 | 1 (0.7) 1 |
| Dizziness | 0 | 0 | 1 (0.9) 1 | 0 | 0 |
| Dysgeusia | 0 | 0 | 0 | 0 | 1 (0.7) 1 |
| Headache | 0 | 1 (3.0) 1 | 5 (4.4) 5 | 2 (5.3) 2 | 4 (2.9) 4 |
| **Psychiatric disorders** | **0** | **1 (3.0) 1** | **0** | **0** | **0** |
| Attention-deficit/ hyperactivity disorder | 0 | 1 (3.0) 1 | 0 | 0 | 0 |
| **Renal and urinary disorders** | **0** | **0** | **0** | **0** | **1 (0.7) 1** |
| Albuminuria | 0 | 0 | 0 | 0 | 1 (0.7) 1 |
| **Reproductive system and breast disorders** | **0** | **0** | **1 (0.9) 1** | **0** | **2 (1.4) 2** |
| Menopausal symptoms | 0 | 0 | 1 (0.9) 1 | 0 | 0 |
| Irregular menstruation | 0 | 0 | 0 | 0 | 2 (1.4) 2 |
| **Respiratory, thoracic, and mediastinal disorders** | **0** | **0** | **0** | **0** | **1 (0.7) 1** |
| Epistaxis | 0 | 0 | 0 | 0 | 1 (0.7) 1 |
| **Skin and subcutaneous tissue disorders** | **1 (1.9) 1** | **0** | **1 (0.9) 1** | **1 (2.6) 1** | **2 (1.4) 3** |
| Acne conglobata | 0 | 0 | 0 | 0 | 1 (0.7) 1 |
| Eczema | 0 | 0 | 0 | 0 | 1 (0.7) 1 |
| Hand dermatitis | 1 (1.9) 1 | 0 | 0 | 0 | 0 |
| Psoriasis | 0 | 0 | 0 | 1 (2.6) 1 | 0 |
| Rash | 0 | 0 | 0 | 0 | 1 (0.7) 1 |
| Rash erythematous | 0 | 0 | 1 (0.9) 1 | 0 | 0 |
| **Vascular disorders** | **0** | **0** | **0** | **1 (2.6) 1** | **0** |
| Hot flash | 0 | 0 | 0 | 1 (2.6) 1 | 0 |

**Supplementary Table 5.** TEAEs by number of sebetralstat administrations.

^a^System organ class and preferred term coded using MedDRA, version 26.0.

E, number of events; MedDRA, Medical Dictionary for Regulatory Activities; n, number of participants with at least 1 adverse event; TEAE, treatment-emergent adverse event.

| **TEAE, number of participants (%), E^a^** | **Sebetralstat** | | | | **Placebo**  **1 or 2 administrations** |
| --- | --- | --- | --- | --- | --- |
|  | **300 mg** | | **600 mg** | |  |
|  | **1**  **administration** | **2 administrations** | **1**  **administration** | **2 administrations** |  |
| **Mucosal attacks** | **(n=23)** | **(n=13)** | **(n=45)** | **(n=17)** | **(n=58)** |
| **Any TEAE** | 4 (17.4) 4 | 3 (23.1) 3 | 9 (20.0) 14 | 5 (29.4) 6 | 15 (25.9) 19 |
| Treatment-related TEAE | 1 (4.3) 1 | 0 | 1 (2.2) 1 | 1 (5.9) 1 | 2 (3.4) 2 |
| **Any TEAE of grade ≥3** | 0 | 0 | 0 | 0 | 0 |
| **Any serious TEAE** | 0 | 0 | 2 (4.4) 2 | 0 | 0 |
| Any treatment-related serious TEAE | 0 | 0 | 0 | 0 | 0 |
| **Any TEAE leading to hospitalization** | 0 | 0 | 2 (4.4) 2 | 0 | 0 |
| **Peripheral attacks** | **(n=28)** | **(n=20)** | **(n=69)** | **(n=21)** | **(n=80)** |
| **Any TEAE** | 4 (14.3) 7 | 5 (25.0) 5 | 11 (15.9) 15 | 3 (14.3) 4 | 9 (11.3) 15 |
| Treatment-related TEAE | 1 (3.6) 1 | 0 | 3 (4.3) 3 | 1 (4.8) 2 | 4 (5.0) 5 |
| **Any TEAE of grade ≥3** | 1 (3.6) 1 | 0 | 0 | 0 | 0 |
| Any treatment-related TEAE of grade ≥3 | 0 | 0 | 0 | 0 | 0 |
| **Any serious TEAE** | 1 (3.6) 1 | 0 | 0 | 0 | 0 |
| Any treatment-related serious TEAE | 0 | 0 | 0 | 0 | 0 |
| **Any TEAE leading to hospitalization** | 1 (3.6) 1 | 0 | 0 | 0 | 0 |

**Supplementary Table 6.** Summary of TEAEs by number of sebetralstat administrations and attack location.

^a^System organ class and preferred term coded using MedDRA, version 26.0.

E, number of events; MedDRA, Medical Dictionary for Regulatory Activities; n, number of participants with at least 1 adverse event; TEAE, treatment-emergent adverse event.

*
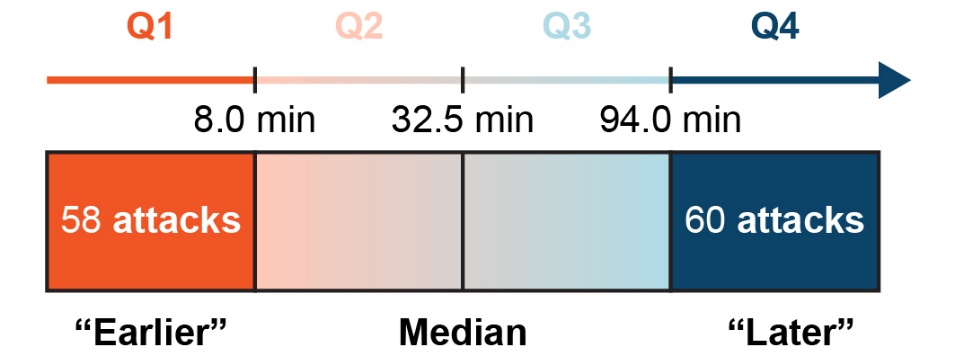
*

**Supplementary Figure 1.** Quartiles for time from HAE attack onset to treatment.

HAE, hereditary angioedema; Q, quartile.


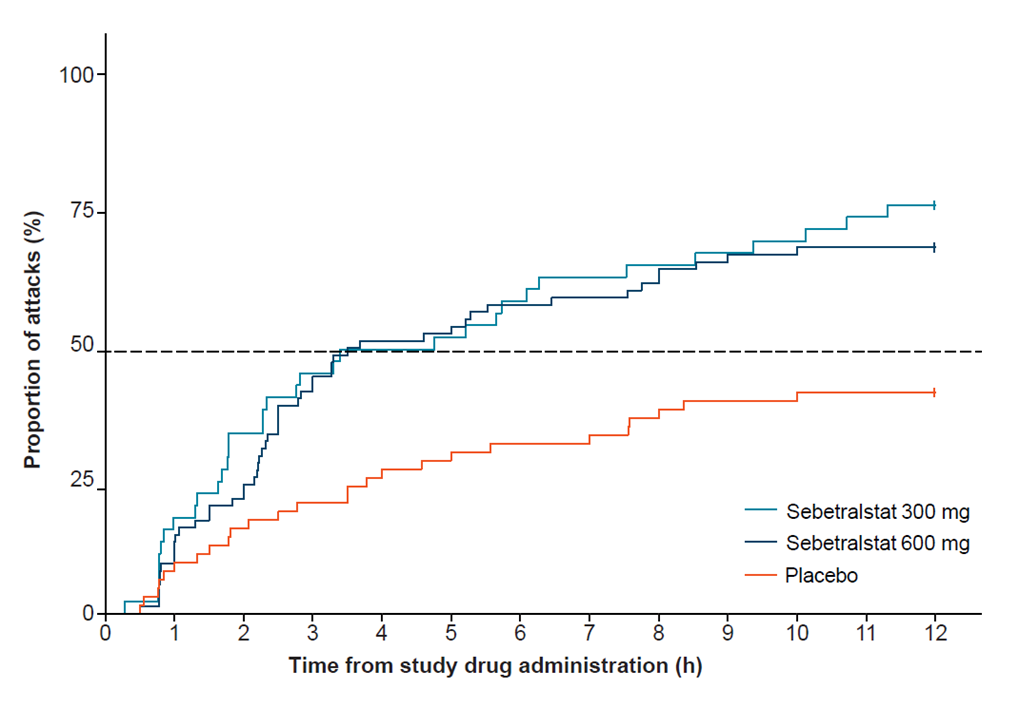


**Supplementary Figure 2.** Time to PGI-S rating of “Mild” for HAE attacks rated “Moderate” or worse at baseline.

h, hours; HAE, hereditary angioedema; PGI-S, Patient Global Impression−Severity.

**Supplementary Figure 1.** Quartiles for time from HAE attack onset to treatment.
HAE, hereditary angioedema.

**Supplementary Figure 2.** Time to PGI-S rating of “Mild” for HAE attacks rated “Moderate” or worse at baseline.
HAE, hereditary angioedema; PGI-S, Patient Global Impression−Severity.
